# Wild fish use visual cues to recognize individual divers

**DOI:** 10.1098/rsbl.2024.0558

**Published:** 2025-02-19

**Authors:** Maëlan Tomasek, Katinka Soller, Alex Jordan

**Affiliations:** ^1^ Behavioural Evolution Research Group, Max Planck Institute of Animal Behaviour, Konstanz 78467, Germany; ^2^ LAboratoire de Psychologie Sociale et COgnitive, UMR6024, CNRS, UCA, Clermont-Ferrand 63000, France

**Keywords:** wild animals, human–animal interactions, recognition, operant conditioning

## Abstract

Many animal species have been shown to discriminate between individual humans in captive settings and may use a variety of cues to do so. Empirical evidence remains scarce for animals in the wild, however, particularly in aquatic contexts. For the first time, we investigated discrimination of individual humans by fish in the wild. We first trained two species of fish, saddled sea bream *Oblada melanura* and black sea bream *Spondyliosoma cantharus*, to follow a human diver to obtain a food reward. We then investigated whether they could discriminate between two human divers and follow the correct one in an operant-conditioning paradigm. We show that both species were able to quickly learn to discriminate between the two divers when they wore different diving gear. However, they showed no preference when both divers wore identical gear, suggesting that discrimination is based predominantly on visual cues from the dive gear. We discuss the implications of these results for ethical considerations and research practices.

## 1. Introduction

Relationships among humans and companion animals have been a cornerstone of human culture through millennia [1]. For pets, livestock and even wild animals that live in close contact with humans, association with humans may confer advantages in terms of increased access to food resources [2], acquisition of information via social learning mechanisms [3] or positive emotional responses [4]. On the other hand, human presence can also be associated with a disadvantageous or potentially dangerous situation. For many of these features, a generalized rule of attraction or repulsion to humans is sufficient, but in other cases, recognition of specific individuals may be required and acquired through experience, for instance if different humans can lead to either positive or negative experiences for the animal. While much of the evidence for animal recognition of humans is anecdotal, there is increasing empirical evidence that certain domestic species [5–9] as well as some non-domestic species living in captivity [7,10] can recognize attributes of individual humans. For wild species less evidence is available, but studies have shown that corvids can discriminate between humans wearing artificial masks [11–13], and that prairie dogs can also discriminate between humans wearing shirts of different colours and encode this information in their alarm calls [14].

While these examples are compelling, they do not demonstrate that animals possess a specific capacity for human recognition, and it remains unclear how animals differentiate among individuals across species boundaries. Indeed, given the energetic and space constraints on the nervous system [15,16], it is more plausible that existing cognitive traits or sensory capacities, evolutionary selected for in other domains or tasks, are co-opted to distinguish among humans. Thus, rather than specific capacity to differentiate humans having been selected, we must ask whether and how animals might use existing capacities to recognize humans. To this end, aquatic species can prove extremely useful as they do not share common human habitats and have limited interactions with humans in underwater environments, minimizing the effect of past experience or selective domestication effects. Perhaps because of the lack of opportunity, evidence for recognition of humans in aquatic systems is rare, but anecdotal evidence from captive conditions suggests that many species may recognize individual humans [17]. Empirically, laboratory studies have demonstrated that captive-bred archerfish could discriminate between images of human faces in an operant-conditioning paradigm, even when the faces were rotated [18], and that wild octopus brought into captivity showed different reactions to two humans associated with positive or negative experience [19]. In both these cases, however, animals were taken out of their natural habitats and exposed to humans (or their pictures) in constrained settings.

Here, we specifically test for the ability of wild teleost fish to recognize humans in their own natural habitat. We study two species of teleosts in the Mediterranean Sea: saddled sea bream *Oblada melanura* and black sea bream *Spondyliosoma cantharus* in open water contexts, where fish were able to voluntarily join or leave an operant-conditioning paradigm. In these experiments, we test (i) whether fish can learn to follow a human diver to obtain a food reward, (ii) whether these fish can discriminate among individual divers and (iii) which cues they use to do so.

## 2. Material and methods

### (a) Field site and subjects

The study site was in the Mediterranean Sea, at the STARESO research station in Corsica, France (42°34'47.8" N, 8°43'28.6" E). Multiple divers occur each day at this site so animals are likely habituated to human presence. Two species voluntarily took part in our experiments: saddled sea bream *O. melanura* and black sea bream *S. cantharus*. Both are common pelagic Mediterranean species and are mostly known for being fished and angled [20]. All individuals were young adults, judging by their size and presence at inshore locations. Across experiments, we were able to individually identify one saddled bream and five black bream based on visual cues (size, colour hue and body markings). Experiments were conducted, while scuba diving between 4 and 8 m, and two diving sessions were conducted per day (one between 08.00 and 10.00 and the other between 14.00 and 16.00) between 12 July and 5 August 2024. An external clinical examination of all fish was conducted by a trained veterinarian (M.T.) and revealed no obvious health issues.

### (b) Training

The training lasted for 12 days, for a total of 23 dives. No fish was observed to spontaneously follow divers before the training. In the beginning of the training, one experimenter (K.S.) settled for 5 min at a starting point (easily identifiable by an anchor underwater; see electronic supplementary material, video S1) and distributed a small food reward (pieces of shrimp) to the fish. Then, she swam away in one direction for about 50 m, at which distance the starting point was not visible anymore. The direction was haphazardly chosen for each dive. After 50 m, the diver once again fed any fish that had followed her. At the beginning of the training, the experimenter produced numerous visual, behavioural and olfactory cues; she was kneeling down in a characteristic position at the starting point, she wore a red rash vest over her wetsuit, carried a black mesh-bag and the zip-lock bag that contained the food reward was pierced to let potential smell out. Progressively, each of these cues was removed until fish followed the experimenter wearing typical dive gear and hovering at the starting point without providing a food reward. At the end of the training, on average eight saddled bream and five black bream followed the experimenter. Of specific individuals, the saddled bream (Bernie) was first identified at dive 5 of the training, four black bream at dives 12 (Left Hump), 15 (Kasi), 19 (Alfi), 21 (Julius) and the last black bream (Geraldine) on the first session of experiment 1. Note that this marks the moment from which we were able to reliably identify them (i.e. identify with absolute certainty at each apparition from one dive to the next) but that they most likely appeared several days prior to this.

### (c) Experiments

**Figure 1.**
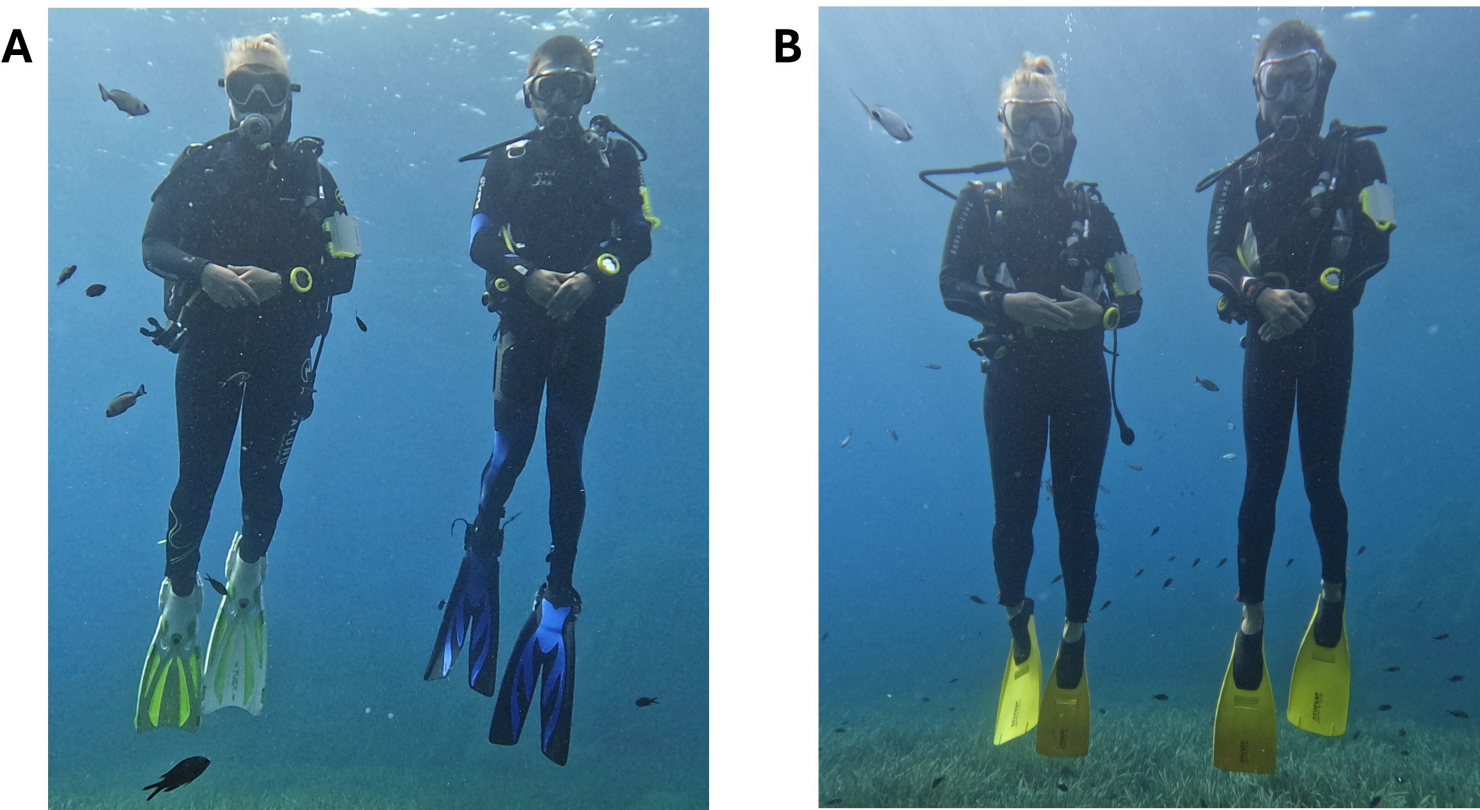
The two divers wearing (A) different dive gear (experiment 1) and (B) identical dive gear (experiment 2).

Our experimental paradigm assessed whether wild fish could learn to follow one of the two divers, who were of similar physical stature, but otherwise differed in their external appearance (figure 1). One diver (K.S.) was always the correct choice, giving a food reward to fish who followed, and the other experimenter (M.T.) was always the incorrect choice. Both carried food in a closed zip-lock bag hidden in a pocket of their diving vests. Experimental sessions began with the two divers swimming to the starting point and hovering for 3 min, after which experimenters swam away in orthogonal directions to one another until an ending point 50 m away where fish received a food reward or not depending on whom they had followed (see electronic supplementary material, video). The same two swimming directions and ending points were used in all trials. After 3 min, the experimenters went back to the starting point, ‘mixed’ themselves by swimming in circles around one other and hovered for 2 min before starting another trial and swimming away in different directions. Each session was composed of three trials. For each experiment, we conducted 10 sessions over the course of 5 consecutive days, for a total of 30 trials per experiment. Swimming directions were assigned randomly before every session, no diver went to the same ending point more than twice in a row and both divers went to both ending points an equal number of trials. Each trial was video recorded, and we counted the number of individuals following each diver as well as monitoring the presence or absence of six specific individuals that could be identified. An individual was considered to follow when it was present at the starting point and followed the diver until the ending point. Individuals that arrived along the way or at the ending point were not counted. In the first experiment, the experimenters wore different dive gear: different fins, masks, buoyancy control devices (BCDs) and wetsuits (figure 1A). In the second experiment, the experimenters wore identical dive gear (figure 1B).

### (d) Statistics

For each experiment, we first assessed discrimination and learning abilities at the population level. We divided trials into three trial epochs: early trials (from trial 1 to 10), middle trials (from 11 to 20) and late trials (from 21 to 30). For both experiments, we analysed the number of fish following each diver using generalized linear mixed-effect models with negative binomial distributions (and not Poisson distributions due to heteroskedasticity; package ‘glmmTMB’ in R [21]). Our predictors of interest were the diver followed, the trial epoch and the species. We also included the direction swam by the divers and the time of day as potential confounders to account for location or time biases that could influence the number of fish present, as well as the session number (from 1 to 10) and the trial number within a given session (from 1 to 3) as random effects. The final model was (Count of fish ~ Diver × Trial epoch × Species + Direction + Time of day + (1|Session) + (1|Trial)). We analysed the residuals to explore the fit of the models (‘DHARMa’ package in R; [22]). We then ran a type II analysis of variance to analyse the contribution of each factor and their interactions (package ‘car’ in R; [23]). Finally, we ran least-square means *post hoc* tests to analyse in each species whether fish had learnt to discriminate between the two divers across trial epochs (function ‘lsmeans’, ‘lsmeans’ package in R; [24]).

We then analysed performances at the individual level for one saddled bream and five black bream. For each individual, we computed a learning index which was, for each trial, +1 if the fish followed the correct diver and −1 if it followed the incorrect diver. Note that all individuals were not always present for all trials. We then ran a generalized linear mixed-effect model (Choice ~ Individual – 1 + Direction + Time of day + (1|Session)) with a binomial distribution. Residuals were analysed as discussed above. We thus could know whether some individuals showed a significant preference for following one of the two divers across all trials.

## 3. Results

**Figure 2.**
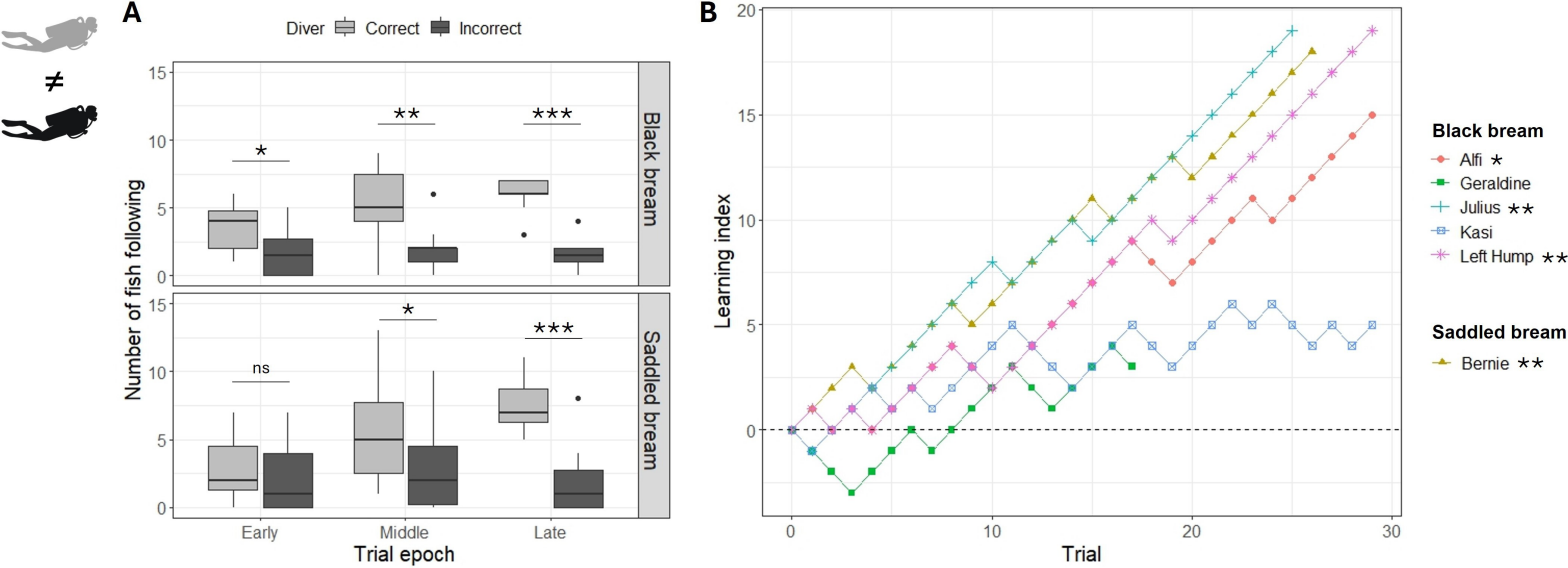
Results of experiment 1, operant-conditioning between two divers wearing different dive gear. (A) Counts of fish following each diver at the population-level across trial epochs (early trials 1−10, middle trials 11−20 and late trials 21−30; least-square means post hoc tests from a generalized linear mixed model (GLMM) with negative binomial distribution, see main text, n.s. = non-significant, *p < 0.05, **p < 0.01 and ***p < 0.001). (B) Learning curves of specific individuals. The learning index increased by +1 if the fish chose the correct diver and decreased by −1 if it chose the incorrect one. Significance signs next to names represent individuals which chose the correct diver significantly more often than the incorrect one across all trials (GLMM with binomial distribution, see main text and electronic supplementary material).

In the first experiment, we assessed the ability of fish to discriminate between two divers wearing different dive gear. At the population level, more saddled bream followed the correct diver than the incorrect one from the middle epoch onwards, and more black bream followed the correct diver from early trials onwards already (figure 2A, electronic supplementary material, table S1). Of the fish that could be individually identified, four individuals (one saddled bream and three black bream) showed increasing learning curves and significantly chose the correct diver more than the incorrect one. The two remaining black bream showed a non-significant learning curve with a positive trend (figure 2B, electronic supplementary material, table S2).

**Figure 3.**
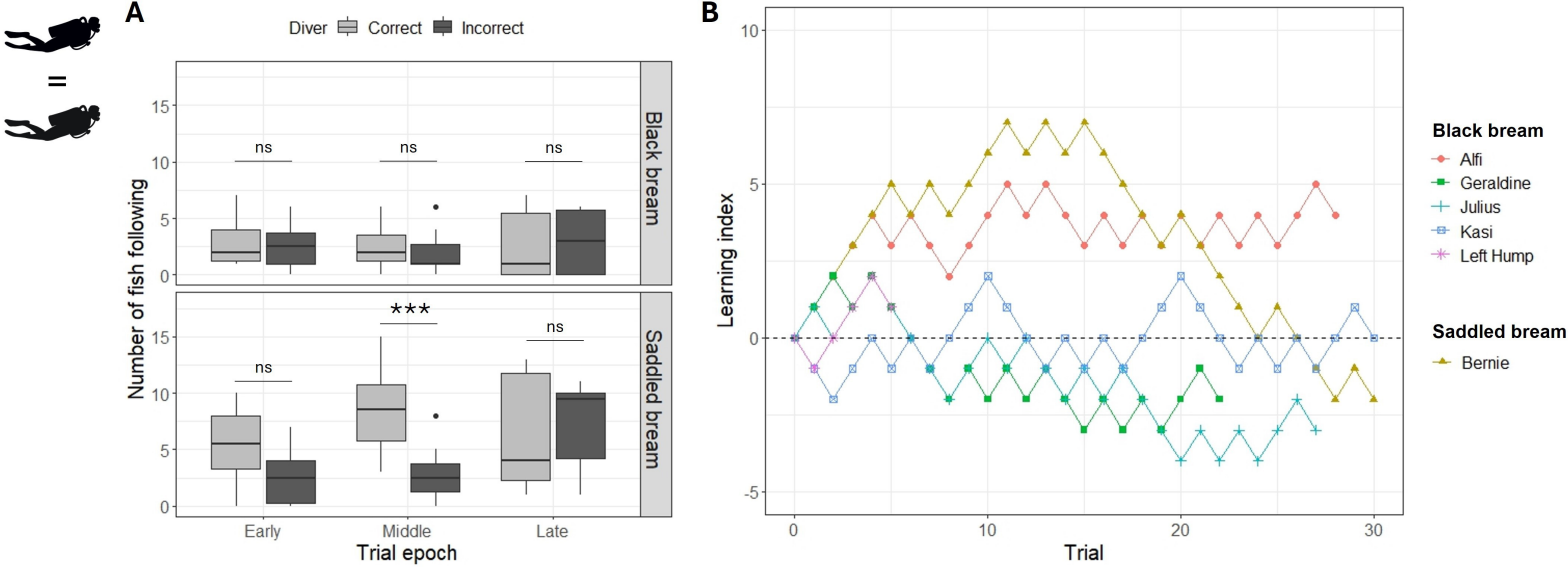
Results of experiment 2, operant-conditioning between two divers wearing identical dive gear. (A) Counts of fish following each diver at the population level (least-square means post hoc tests from a generalized linear mixed model (GLMM) with negative binomial distribution, see main text, n.s. = non-significant, ***p < 0.001). (B) Learning curves of specific individuals. The learning index increased by +1 if the fish chose the correct diver and decreased by −1 if it chose the incorrect one. No individual chose the correct diver significantly more than the incorrect one across all trials (GLMM with binomial distribution, see main text and electronic supplementary materiall).

In the second experiment, we assessed discrimination between two divers wearing identical dive gear. At the population level, black bream did not follow one diver more than the other, and saddled bream followed the correct diver significantly more often in middle epoch but not in early and late epochs (figure 3A, electronic supplementary material, table S3). At the individual level, no individual chose the correct diver more than the incorrect one (figure 3B, electronic supplementary material, table S4).

## 4. Discussion

In this study, we showed that saddled sea bream *O. melanura* and black sea bream *S. cantharus* could be trained to follow specific human divers who differed in their external appearance. By conducting this study with wild fish, we avoided any confounding effects of selective domestication or prior experience with humans [25,26]. This provided strong evidence that pre-existing capacities, rather than a specific mechanism to recognize humans, were being employed by the participants of our study. This effect was observed both at the population level and within identifiable individuals. However, when divers wore identical dive gear, correct identification of divers was greatly diminished, suggesting that discrimination was based predominantly on visual cues. Like almost all teleost fish, the participants of our study are tetrachromatic and possess acute colour vision [27]. Moreover, colour discrimination has been observed in feeding domains in related species, further supporting an existing capacity for colour discrimination having been co-opted for distinguishing humans in our experiments [28].

While some fish species can focus on detailed patterns to recognize individual conspecifics (e.g. facial colour patterns in cichlids; [29]), individual recognition might be more difficult in heterospecifics, especially in species as different as humans. Some animals can discriminate humans even though they are wearing identical outfits [7–9,19] and archerfish can discriminate images of human faces [18], but sea bream were not able to distinguish between the divers with identical gear. Very few visual differences remain once two divers dress identically due to obstruction by the gear itself. Only hair, beard, hands and to a degree body morphology remain visible; faces are not as reliable as on land because they are distorted by diving equipment. Our results show that fish can use dive gear to correctly identify divers, but did not use other subtle differences to distinguish them. It is worth considering the parallels with human divers, who also readily use equipment to identify one another when other visual cues are absent or when diving with unfamiliar divers. Further experiments could confirm the role of the dive gear and investigate which specific components might be used by the fish by using more subtle changes in diver appearance.

Because our study was conducted in the wild, we observed fluctuations in the number of fish that participated in the experiments. Generally, the number of fish increased across trials and experiments, especially for saddled bream as participation of black bream in the second experiment decreased. We could not reliably link these fluctuations with notable environmental or behavioural variations. As a consequence, we cannot be certain of the exact number of individuals who learnt to discriminate between the two divers. Indeed, some individuals may have followed other fish rather than the diver, especially naive individuals that arrived after the start of the study and which we could not monitor. Both species are grouping species, and social attraction to other fish may have contributed to their response [30]. However, we can be confident that several individuals have learnt to discriminate and that decisions did not rely solely on social attraction. Indeed, fish almost never followed only one diver, which may be expected under conditions of strong social attraction, and in some cases a single individual would follow a diver. We also observed different strategies in the specific individuals, for instance one black sea bream (Kasi) chose to always go to the same direction in the second experiment, regardless of diver identity, while other fish continued to go to both directions.

The fact that wild bream can discriminate between divers adds scientific evidence to the numerous accounts suggesting differentiated relationships between fish and specific humans [17]. Individual discrimination is required for specific bonds between humans and non-human animals, and our results suggest that this possibility should also be considered in taxa such as fish. Indeed, this ‘inevitable bond’ also has implications for research, influencing for instance how animals will react to different experimenters [31]. Cognitive studies in fish are rarely conducted in the wild due to practical difficulties [32], but our study demonstrates that certain species can willingly engage with humans over a relatively short time frame. Such species are amenable to cognitive research as the same individuals will repeatedly interact with individual divers, an ideal configuration to offer cognitive tasks that require repeated iterations for learning. Our study thus encourages a reappraisal of the methodological avenues to study cognitive abilities of wild fish under natural conditions. It also demonstrates a potential difficulty when conducting such experiments that could be disturbed by fish following specific experimenters. Researchers might not always want to be followed all around by fish, but if they do, they will not be disappointed.

## Data Availability

Data and R script are available as electronic supplementary material. A description of the dataset is present on the first page of the data file. Supplementary material is available online [33].
